# Left main coronary artery stenosis assessment on coronary CT angiography

**DOI:** 10.1016/j.ejro.2026.100803

**Published:** 2026-07-30

**Authors:** Mahdi Zahedi, Pooya Eini, Mohammad Javad Alemzadeh-Ansari, Marzieh Motevalli, Amirafraz Fallah Najmabadi, Golnaz Houshmand

**Affiliations:** a Cardiovascular Imaging Research Center, Rajaie Cardiovascular Institute, Tehran, Iran; b Ischemic Disorders Research Center, Golestan University of Medical Sciences, Gorgan, Iran; c Cardiovascular Intervention Research Center, Rajaie Cardiovascular Institute, Tehran, Iran

**Keywords:** Left Main Coronary Artery, Coronary CT Angiography, Intravascular Ultrasound, Minimum Lumen Area, Coronary Artery stenosis

## Abstract

**Background:**

Assessment of Left Main Coronary Artery (LMCA) stenosis is critical due to its prognostic significance. LMCA cross-sectional area in Coronary Computed Tomography Angiography (CCTA) can provide a non-invasive alternative, but data on its correlation with IVUS remain limited, particularly in different ethnic groups. This study aimed to evaluate CT LM-CSA in determining significant left main stenosis compared to IVUS-MLA in Iranian patients.

**Methods:**

We included patients who underwent IVUS for LM stenosis evaluation and had CCTA performed within a maximum of 90 days before IVUS. LM-CSA measured at the narrowest segment on CCTA and MLA obtained via IVUS. Correlation assessed using Pearson’s coefficient; agreement evaluated with Bland–Altman analysis. Receiver operating characteristic (ROC) analysis determined the optimal LM-CSA cut-off.

**Results:**

Forty-five patients were included (mean age 59.2 ± 10.1 years; 60% male). IVUS-defined significant LMCA stenosis (MLA <6.0 mm²) was present in 33.3% patients. Baseline characteristics were similar between groups. CCTA-derived LM-CSA showed a strong correlation with IVUS-derived MLA (r = 0.93, p < 0.001). A CT-derived LM-CSA cut-off of 6.2 mm² identified for predicting significant stenosis, yielding 95.6% diagnostic accuracy and an AUC of 0.94 (95% CI: 0.86–1.00), with sensitivity of 88.2% (95% CI: 63.6–98.5%), specificity of 100% (95% CI: 87.7–100%), positive predictive value of 100% (95% CI: 78.2–100%), and negative predictive value of 93.3% (95% CI: 77.9–99.2%).

**Conclusions:**

CCTA LM-CSA is a reliable non-invasive metric for LMCA stenosis assessment. The optimized cut-off provides a threshold derived in an Iranian cohort that requires further validation before it can be used to guide deferral of invasive procedures.

## Highlights

• CCTA-derived LM-CSA showed strong correlation with IVUS -MLA for assessment of LMCA stenosis.
• A CT-derived LM-CSA cut-off of 6.2 mm² demonstrated high diagnostic performance for identifying significant LMCA stenosis in this Iranian cohort.
• These findings are exploratory and support the potential role of CCTA as a non-invasive tool for LMCA assessment.
• Larger prospective studies are needed before routine clinical implementation of CCTA for LMCA assessment.

## 1 Introduction

Coronary Artery Disease (CAD) remains a leading cause of mortality globally. Left Main Coronary Artery (LMCA) stenosis is of vital importance, as this vessel is responsible for supplying blood to approximately 70–100% of the left ventricular myocardium [1]. The presence of significant stenosis in the LMCA is associated with a markedly poor prognosis and increased risk of mortality [2]. A precise and reliable evaluation of LMCA anatomy and pathology is both a challenge and an essential requirement in modern interventional cardiology. For several decades, Invasive Coronary Angiography (ICA) was accepted as the gold standard for the diagnosis of coronary stenosis [3]. However, the visual assessment of the LMCA using conventional angiography is complicated by inherent limitations. The two-dimensional nature of angiography, the phenomenon of vessel overlap (superimposition) due to the artery’s bifurcation, and significant anatomical variation often lead to angiographic ambiguity. [4] This can result in inaccurate lesion assessment, causing both overestimation (potentially leading to unnecessary, high-risk intervention) and underestimation (withholding necessary, life-saving intervention), both of which have serious adverse clinical outcomes.

To overcome the shortcomings of ICA, Intravascular Ultrasound (IVUS) emerged as a superior intravascular imaging technology. IVUS provides a high-resolution, cross-sectional view of the artery, enabling accurate quantitative and qualitative analysis of lumen size, plaque characteristics, and extent, independent of the viewing angle [5]. Previous meta-analyses have affirmed the strong prognostic value of the Minimum Lumen Area (MLA) measured by IVUS in the LMCA [6], [7]. Accordingly, an MLA value of less than 6.0 mm² has been universally established as the definitive cut-off for defining functionally significant LMCA stenosis, serving as the gold standard for guiding revascularization decisions [8], [9]. Simultaneously, Coronary Computed Tomography Angiography (CCTA) has advanced as an accurate, non-invasive method for diagnosing CAD. Technological improvements have enabled CCTA to provide high-quality quantitative data and its accuracy in measuring vessel areas has been confirmed in several studies compared with IVUS in proximal coronary segments [10]. The measurement of the Left Main Cross-Sectional Area (LM-CSA) by CCTA offers a simple and accessible non-invasive anatomical index for initial assessment. Despite existing evidence supporting the general accuracy of CCTA, a critical knowledge gap persists regarding the direct, quantitative correlation between the CCTA parameter (LM-CSA) and the IVUS gold standard for predicting significant LMCA stenosis.

There are limited data available on the diagnostic performance of CCTA compared to IVUS for LMCA evaluation specifically in different ethnicity. Given known ethnic variability in LMCA dimensions, extrapolating Western CT-derived thresholds to Middle Eastern populations may be inappropriate. Therefore, the primary objective of this study was to determine the correlation between the LM-CSA measured by CCTA and the MLA measured by IVUS. The secondary objective was to determine the optimal diagnostic cut-off value for LM-CSA in CCTA for predicting functionally significant LMCA stenosis (IVUS-MLA < 6.0 mm²), and to calculate the resulting sensitivity, specificity, and predictive values.

## 2 Methods

### 2.1 Study design and population

Between January 2016 and December 2025, 200 consecutive patients underwent both CCTA and invasive coronary angiography at Rajaie Cardiovascular Institute. Of these, 45 patients met all predefined inclusion criteria and formed the final study cohort. Patients were included if they were ≥ 18 years old, had undergone diagnostic-quality CCTA free of severe motion or blooming artifacts, and subsequently underwent IVUS evaluation of the left main coronary artery within 90 days, with no intervening acute coronary syndrome or revascularization.

Exclusion criteria were prior coronary artery bypass grafting, previous left main stent implantation, nondiagnostic CCTA image quality, uncontrolled atrial fibrillation during CT acquisition, absence of IVUS imaging of the LMCA, or incomplete imaging data.

### 2.2 Demographic and clinical data collection

We recorded age, sex, body mass index (BMI), medical history (hypertension, diabetes mellitus, dyslipidemia), smoking status, family history of premature coronary artery disease, Laboratory measurements included hemoglobin (HB), serum creatinine (Cr), blood urea nitrogen (BUN), triglycerides (TG), high-density lipoprotein cholesterol (HDL), low-density lipoprotein cholesterol (LDL), and fasting blood sugar (FBS) through patient interviews and review of electronic medical records.

### 2.3 CCTA protocol

CCTA was performed using multi-detector CT scanners (256-slice or 384-slice dual-source systems). The imaging protocol followed standard guidelines of the European Society of Cardiology and the Society of Cardiovascular Computed Tomography. To optimize image quality, heart-rate-lowering medications such as metoprolol and sublingual vasodilators such as nitroglycerin were administered when indicated.

Image analysis was performed on a dedicated workstation equipped with advanced cardiac analysis software (Syngo.via). CT-derived lumen cross-sectional area (LM-CSA) was measured by manual planimetry at the narrowest luminal segment on images reconstructed during mid to end diastole (typically 70–80% of the R–R interval) in order to minimize cardiac motion artifacts. Measurements were performed perpendicular to the vessel centerline at standardized distances from the ostium and bifurcation when anatomically feasible. Image quality was assessed on a 4-point scale (1: uninterpretable to 4: excellent), and low-quality images were excluded. All CCTA measurements were performed by two experienced imaging cardiologists who were blinded to the IVUS results. Interobserver agreement was excellent for CCTA-derived LM-CSA measurements (ICC >0.90), supporting the reproducibility of the measurement protocol.

### 2.4 IVUS protocol

The procedure was performed using commercially available systems (Boston Scientific or Terumo). The MLA was identified and measured at the narrowest segment of the Left Main artery. A significant stenosis was defined as a MLA < 6.0 mm². An experienced interventional cardiologist, blinded to the CCTA findings, performed all quantitative measurements using dedicated IVUS analysis software.

### 2.5 Sample size and statistical analysis

All statistical analyses used STATA version 18. The sample size calculation targeted a two‑sided correlation test. The sample size calculation aimed to detect a moderate‑to‑strong correlation (assuming an effect size of 0.50, a type I error rate (alpha) of 0.05, and a power of 0.95 (beta = 0.05)). Based on these assumptions, a minimum sample size of 45 patients met the requirement.

Continuous variables were summarized as mean (SD) or median (IQR), while categorical variables were reported as frequencies and percentages. Group comparisons were performed using independent-sample T-tests for continuous variables and chi-square tests for categorical variables. The association between IVUS and CCTA measurements was examined using Pearson’s correlation coefficient, and agreement between the two modalities for LMCA-MLA was evaluated using Bland–Altman analysis. Diagnostic performance was assessed using ROC analysis to determine the ability of CCTA to identify an IVUS-MLA < 6.0 mm². An optimal CCTA threshold was derived using the Liu index. This index provides a balanced optimization of sensitivity and specificity, which is appropriate in settings where both false positive and false negative classification of left main disease may have important clinical consequences. Sensitivity, specificity, positive predictive value (PPV), and negative predictive value (NPV) were calculated for this threshold. To explore potential contributors to false-positive CCTA findings, clinical variables including age, smoking status, hypertension, diabetes mellitus, history of transient ischemic attack, family history of cardiovascular disease, dyslipidemia, chest pain, and dyspnea were examined. Statistical significance was defined as a two-sided p-value < 0.05.

## 3 Results

### 3.1 Study flow and patient selection

**Fig. 1:**
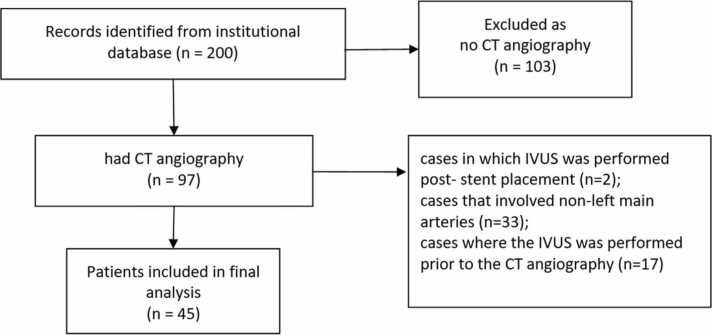
study flow diagram outlining patient selection and reasons for exclusion.

A total of 200 IVUS procedure records were initially identified from the institutional database. Among these, 97 patients had also undergone CT angiography. The following were excluded: 2 cases in which IVUS was performed post- stent placement; 33 cases that involved non-left main arteries; and 17 cases where the IVUS was performed before the CT angiography. Finally, 45 patients with diagnostic quality CCTA and corresponding IVUS evaluation of the native LMCA were included in the analysis. (Fig. 1).

### 3.2 Patient characteristics

The mean age was 59.2 ± 10.1 years (median 58, IQR 13), and 27 patients (60.0%) were male. The most common presenting symptom was chest pain (n = 35, 77.8%). Statin therapy was common, with Atorvastatin 40 mg being the most frequently used regimen (n = 25, 55.6%). Cardiovascular risk factors included hypertension (n = 15, 33.3%), diabetes mellitus (n = 8, 17.8%), current or previous smoking (n = 6, 13.3%), dyslipidemia (n = 16, 35.6%), and family history of coronary artery disease (n = 10, 22.2%). Laboratory parameters included mean hemoglobin 13.0 ± 1.5 g/dL (median 13.5, IQR 2.0), creatinine 1.0 ± 0.2 mg/dL (median 1.0, IQR 0.2), blood urea nitrogen 14.9 ± 4.0 mg/dL (median 14, IQR 5), triglycerides 135 ± 42 mg/dL (median 133, IQR 76.5), HDL cholesterol 39 ± 17 mg/dL (median 37, IQR 15), LDL cholesterol 81 ± 38 mg/dL (median 79, IQR 43), and fasting blood sugar 113 ± 22 mg/dL (median 114, IQR 25). Mean ejection fraction was 50.7 ± 5.9% (median 50, IQR 5).

IVUS-derived MLA < 6.0 mm², indicating significant LMCA stenosis, was observed in 15 patients (33.3%). Statin use differed between groups (p = 0.044), with higher-dose rosuvastatin use more frequent among patients with IVUS-defined significant LMCA stenosis. No statistically significant differences were observed in age, sex, ejection fraction, cardiovascular risk factors, presenting symptoms, or laboratory parameters between groups (all p > 0.05). The interval between procedures was 2 days (IQR 1–4) overall, 1 day (IQR 1–4) in non-significant LMCA group, and 2 days (IQR 2–9) in significant LMCA stenosis group. No patient had documented acute coronary syndrome, coronary revascularization, or another major clinical event between the CCTA and IVUS examinations. Baseline clinical and laboratory characteristics stratified by IVUS-defined LMCA stenosis are presented in Table 1.

**Table 1:** Baseline clinical and laboratory characteristics.

Variable	Overall (n = 45)	IVUS non-significant LMCA stenosis MLA ≥ 6 mm² (n = 30)	IVUS significant LMCA stenosis MLA < 6 mm² (n = 15)	p-value
Age, years	59.2 ± 10.1; 58 (13)	58.9 ± 8.8; 58.5 (13)	59.7 ± 12.7; 58 (14)	0.604
Male sex, n (%)	27 (60.0)	17 (56.7)	10 (66.7)	0.748
Ejection fraction, %	50.7 ± 5.9; 50 (5)	50.7 ± 6.2; 55 (5)	50.8 ± 5.3; 50 (5)	0.714
Hemoglobin, g/dL	13.0 ± 1.5; 13.5 (2.0)	13.0 ± 1.5; 13.5 (2.0)	13.1 ± 1.6; 13.9 (2.0)	0.591
Creatinine, mg/dL	1.0 ± 0.2; 1.0 (0.2)	0.95 ± 0.16; 0.9 (0.2)	1.02 ± 0.15; 1.0 (0.1)	0.285
Blood urea nitrogen, mg/dL	14.9 ± 4.0; 14 (5)	15.2 ± 2.8; 14 (5)	14.0 ± 6.8; 13 (10)	0.627
Triglycerides, mg/dL	135 ± 42; 133 (76.5)	141 ± 47; 151 (87)	115 ± 21; 115 (30)	0.505
HDL cholesterol, mg/dL	39 ± 17; 37 (15)	41.6 ± 19.9; 38 (15)	33.5 ± 4.9; 33.5 (7)	0.699
LDL cholesterol, mg/dL	81 ± 38; 79 (43)	72.7 ± 37.4; 75 (34)	111.5 ± 26.2; 111.5 (37)	0.142
Fasting blood sugar, mg/dL	113 ± 22; 114 (25)	110 ± 13; 114 (25)	117 ± 38; 117 (54)	1.000
Statin use, n (%)				**0.044**
Atorvastatin 20 mg	2 (4.4)	1 (3.3)	1 (6.7)	
Atorvastatin 40 mg	25 (55.6)	20 (66.7)	5 (33.3)	
Atorvastatin 80 mg	1 (2.2)	0 (0.0)	1 (6.7)	
Rosuvastatin 10 mg	1 (2.2)	1 (3.3)	0 (0.0)	
Rosuvastatin 20 mg	4 (8.9)	1 (3.3)	3 (20.0)	
Rosuvastatin 40 mg	8 (17.8)	3 (10.0)	5 (33.3)	
No statin use	4 (8.9)	4 (13.3)	0 (0.0)	
Smoking, current/previous, n (%)	6 (13.3)	4 (13.3)	2 (13.3)	1.000
Hypertension, n (%)	15 (33.3)	10 (33.3)	5 (33.3)	1.000
Diabetes mellitus, n (%)	8 (17.8)	4 (13.3)	4 (26.7)	0.410
Transient ischemic attack, n (%)	1 (2.2)	1 (3.3)	0 (0.0)	1.000
Family history of CAD, n (%)	10 (22.2)	8 (26.7)	2 (13.3)	0.456
Dyslipidemia, n (%)	16 (35.6)	9 (30.0)	7 (46.7)	0.331
Chest pain, n (%)	35 (77.8)	22 (73.3)	13 (86.7)	0.456
Dyspnea, n (%)	2 (4.4)	0 (0.0)	2 (13.3)	0.106
Interval between procedures, days	2 (1–4)	1 (1–4)	2 (2–9)	0.06

### 3.3 LMCA measurements and correlation

The mean CCTA-derived LM-CSA was 7.87 ± 3.55 mm² (median 7.85, IQR 5.15), and the mean IVUS-derived MLA was 8.48 ± 4.21 mm² (median 8.5, IQR 5.5). There was a strong positive correlation between IVUS-derived MLA and CCTA-derived LM‑CSA measurements (Pearson's r = 0.93, p < 0.001), which was confirmed by a strong monotonic relationship on nonparametric testing (Spearman’s ρ = 0.88, p < 0.001).

### 3.4 Diagnostic performance of CCTA

**Fig. 2:**
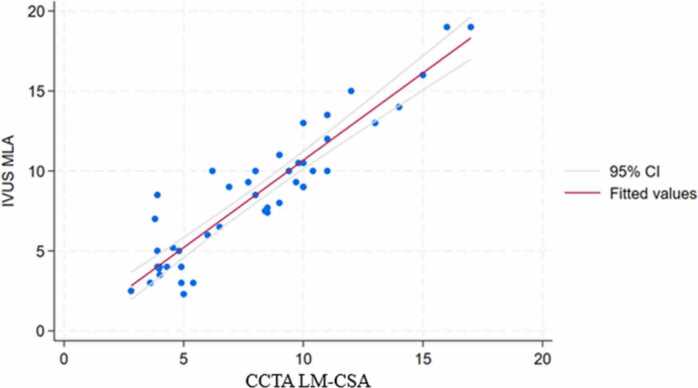
Scatter plot with regression line showing the relationship between CCTA-derived LM‑CSA and IVUS-derived MLA in the left main coronary artery.

**Fig. 3:**
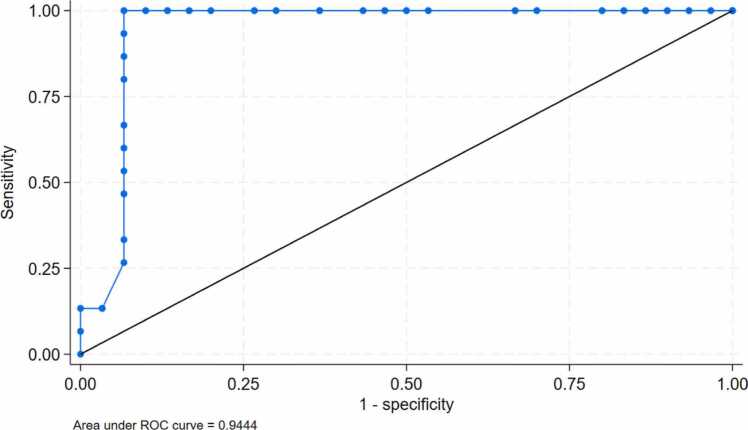
ROC plot for CCTA-derived LM-CSA predicting IVUS MLA < 6 mm² with optimal cut-off.

Using Liu’s method, the threshold of 6.2 mm² maximized the product of sensitivity and specificity (Liu index = 0.88) among all evaluated cut-points. CCTA correctly classified 43 of 45 lesions. This threshold yielded a sensitivity of 88.2% (95% CI: 63.6–98.5%), specificity of 100% (95% CI: 87.7–100%), positive predictive value of 100% (95% CI: 78.2–100%), negative predictive value of 93.3% (95% CI: 77.9–99.2%), and the overall diagnostic accuracy of 95.6% (Fig. 2). The corresponding diagnostic accuracy, expressed as the ROC area, was 0.94 (95% CI: 0.86–1.00) (Fig. 3). Diagnostic performance was stable across adjacent thresholds between 6.0 and 6.5 mm², with minimal trade-off between sensitivity and specificity, supporting the robustness of the selected cut-off. However, given the limited number of patients with significant stenosis, these estimates should be interpreted as exploratory rather than definitive.

### 3.5 Agreement between IVUS and CCTA

**Fig. 4:**
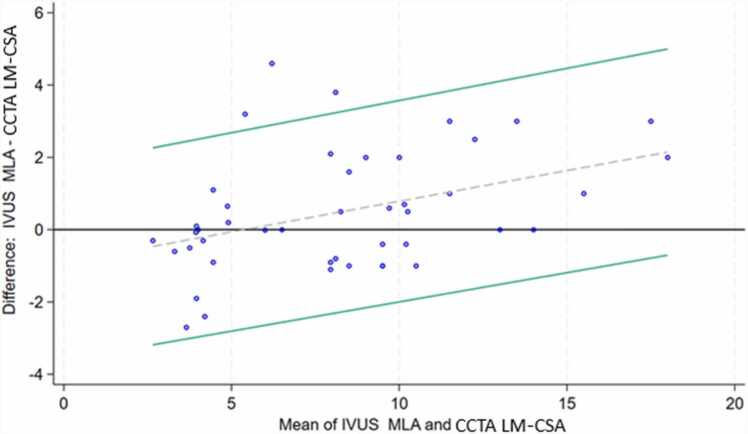
Bland–Altman plot of CCTA LM-CSA vs IVUS MLA agreement.

Bland–Altman analysis revealed a mean difference (IVUS-MLA and CCTA- LM‑CSA) of 0.49 mm² (SD 1.64 mm²), indicating a small systematic underestimation of LM‑CSA by CCTA relative to IVUS-MLA. Regression analysis demonstrated significant proportional bias (slope = 0.17, p = 0.006), with increasing disagreement at higher mean IVUS-MLA values, described by the bias equation: −0.91 + 0.17 × mean IVUS-MLA. No significant trend was observed in the standard deviation of the differences (slope = 0.004, p = 0.930), indicating the absence of heteroscedasticity. Regression-based 95% limits of agreement were −3.61 + 0.16 × mean IVUS-MLA and 1.79 + 0.18 × mean IVUS-MLA (Fig. 4).

## 4 Discussion

In this study, we demonstrate that CCTA-derived LM-CSA provides high diagnostic performance for identifying IVUS-defined significant LMCA stenosis. Using an optimized threshold of < 6.2 mm², CCTA achieved high sensitivity 88.2% (95% CI: 63.6–98.5%), specificity 100% (95% CI: 87.7–100%), and an area under the ROC curve of 0.94 (95% CI: 0.86–1.00), indicating good discriminative ability. These findings support the role of CCTA as a non-invasive modality for anatomical assessment of LMCA disease severity when compared against IVUS as the reference standard. The primary novel contribution of this research is the derivation of a specific, optimized LM-CSA cut-off value derived in CCTA for significant left main stenosis in the Iranian patients as, limited information exists on CCTA's diagnostic performance against IVUS in this demographic. This threshold was derived from a single, modestly sized cohort in which only 15 patients had significant left main stenosis, and it has not undergone internal or external validation. It should therefore be regarded as exploratory and hypothesis-generating, with uncertain generalizability until it is confirmed in larger, independent populations.

From a clinical standpoint, the stability of patients between CCTA and IVUS supports the assumption that the anatomical severity of left main disease remained unchanged during the inter-test interval. The absence of intervening ischemic events or revascularization suggests that measurements obtained by the two modalities reflect the same disease state rather than interval progression, thereby strengthening the validity of the observed agreement and diagnostic performance of CCTA-derived LM-CSA when referenced against IVUS.

Prior studies comparing CCTA-derived LM-CSA with IVUS-MLA have consistently reported strong correlations and clinically meaningful agreement. However, the proposed CCTA thresholds have varied depending on study population, scanner technology, and clinical intent. Earlier work using 64-slice CT demonstrated good correlation between CCTA-derived LM-CSA and IVUS-MLA measurements, with IVUS cut-offs of approximately 6.0 mm² commonly applied to define significant LMCA disease [11]. Our results are closely aligned with this IVUS reference threshold, suggesting that contemporary CCTA techniques can approximate IVUS-based anatomical criteria with a high degree of accuracy.

More recent CCTA studies have proposed higher thresholds to optimize sensitivity and negative predictive value, particularly in the context of safely ruling out significant LMCA stenosis. For example, a CCTA-derived LM-CSA cut-off of < 8.29 mm² was reported to provide high sensitivity and NPV for detecting IVUS-defined significant disease, favoring a conservative rule-out strategy [9]. While such higher thresholds may be appropriate when prioritizing sensitivity, they inevitably reduce specificity and may increase false-positive rates. In contrast, our selected threshold of 6.2 mm² achieves a favorable balance between sensitivity and specificity, resulting in superior overall classification accuracy and perfect specificity in this cohort, which is particularly relevant when CCTA findings may directly influence subsequent invasive management.

The AUC observed in our study compares favorably with prior validation studies and underscores the robustness of CCTA-derived LM-CSA as a continuous diagnostic marker. An AUC approaching 0.95 suggests that CCTA-derived LM-CSA retains high discriminative power across a wide range of stenosis severities, consistent with previous reports demonstrating the utility of quantitative CT metrics for lesion assessment [12]. Importantly, this level of performance was achieved despite the known technical limitations of CCTA, including partial-volume effects and blooming from calcified plaque.

Our Bland–Altman analysis provides additional insight into the relationship between CCTA and IVUS measurements. We observed a small fixed bias, with CCTA modestly underestimating relative to IVUS, as well as evidence of proportional bias, whereby measurement differences increased slightly at higher values. However, the absence of heteroscedasticity indicates that measurement variability remained stable across the range of observed values. Similar patterns of systematic underestimation by CCTA have been reported previously and are generally attributed to differences in spatial resolution and lumen border delineation between CCTA and IVUS [12]. Importantly, the predictable nature of this bias supports the use of modality-specific thresholds rather than undermining the clinical utility of CCTA.

The observed systematic underestimation of lumen area by CCTA may be partially attributable to coronary calcification, particularly in patients with high calcium burden. Extensive calcification can accentuate blooming and beam-hardening artifacts, which obscure the true luminal boundary and may lead to smaller apparent lumen measurements on CT. Although Agatston scores were not available uniformly enough in the present cohort to permit formal subgroup analysis, prior studies have demonstrated that CCTA diagnostic performance is reduced in severely calcified coronary segments and that artifact-reduction techniques can improve specificity [13], [14].

Furthermore, another study evaluated 54 symptomatic, intermediate-risk patients (162 coronary sites) who underwent 64-slice MDCT and IVUS, stratified by coronary artery calcium score (CACS). MDCT showed moderate to strong correlations with IVUS measurements, particularly for lumen area (r = 0.837), with moderate correlations for external elastic membrane area (r = 0.514) and plaque area (r = 0.578). Plaque area measurements remained consistently correlated across all CACS subgroups (r = 0.562–0.671), suggesting that MDCT provides reasonably reliable plaque assessment irrespective of calcium burden [15].

Conversely, in 181 intermediate coronary lesions, CCTA and IVUS measurements were compared with fractional flow reserve (FFR) as the reference for ischemia (FFR ≤ 0.80). CCTA consistently underestimated luminal dimensions compared with IVUS, demonstrating significantly smaller minimal lumen area (2.2 ± 1.2 vs. 3.2 ± 1.2 mm², P < 0.001) across all lesion locations and plaque types. When assessing functional significance, IVUS-derived minimal lumen area showed superior diagnostic performance for detecting ischemia compared with CCTA-derived LM-CSA (AUC 0.801 vs. 0.712, P = 0.03), indicating that although CCTA provides valuable non-invasive anatomical information, IVUS remains more accurate for lesion severity and physiological relevance [16].

Demographic variations also play a critical role; A comparative CCTA study showed that Asians have smaller coronary artery dimensions than Caucasians, especially at the left main coronary artery, even after matching for age, sex, body surface area, and coronary dominance pattern. This difference was most pronounced in the LMCA luminal area [17]. From a technological standpoint, CT-derived FFR (FFR-CT) provides both anatomical and physiological assessment of coronary lesions and typically improves diagnostic performance over anatomy alone, especially for ischemia detection. Studies show FFR-CT can better predict lesion-specific ischemia than CCTA stenosis measurements alone, with higher diagnostic accuracy and ability to reduce unnecessary invasive angiography [18]. However, anatomical CCTA measurements (e.g., minimal lumen area or cross-sectional area) remain essential as the first-line non-invasive evaluation, are broadly accessible, and are usually less costly and more widely available than FFR-CT because FFR-CT requires additional computational analysis [19].

While the correlation was strong, the observed differences between CCTA and IVUS measurements can be attributed to inherent technical and biological factors. CCTA is known to be susceptible to image artifacts, particularly the "blooming artifact" induced by dense coronary calcification, which can obscure the true lumen and lead to measurement bias [14]. Furthermore, the temporal and spatial resolution of CCTA is dependent on factors such as heart rate stability and patient cooperation (e.g., breath-holding) [13]. In contrast, IVUS provides a definitive, high-resolution cross-sectional assessment, making it the least susceptible to these external factors, though it may be subject to minor procedural variations such as catheter selection or pull-back speed. The persistence of a high correlation despite these limitations validates both modalities when their respective technical constraints are considered.

From a clinical perspective, our findings suggest that a CCTA-derived LM-CSA threshold near 6.2 mm² may serve as a practical and evidence-based surrogate for the established IVUS criterion of < 6.0 mm² when evaluating LMCA stenosis. This is particularly relevant in patients in whom invasive assessment is undesirable or carries increased procedural risk. At the same time, our data support the use of a more conservative CCTA threshold for rule-out strategies when maximizing negative predictive value is the primary objective, consistent with prior CCTA studies emphasizing safety in excluding significant disease [9].

This targeted approach allows for rational management of resources and enhanced patient care. Furthermore, ongoing research exploring advanced CCTA parameters, such as the combination of LM-CSA with Percentage Atheroma Volume (PAV), could further enhance the predictive value for high-risk lesions.

### 4.1 Study lihmitations

The study was conducted at a single tertiary care center with small sample size and no external validation cohort which may affect the generalizability and external validity of the derived cut-off value to other international populations. Because eligibility required both diagnostic-quality CCTA and IVUS interrogation of the left main, the final cohort represents a highly selected subgroup drawn from 200 initially screened patients, with substantial exclusions for non-left-main IVUS, imaging timing, and image quality. This selection, together with the partial-verification design in which IVUS was not performed in all patients undergoing CCTA, may have inflated the observed diagnostic performance relative to that expected in routine, unselected clinical populations. Factors such as gender and the extent of vessel calcium can act as confounding factors that influence the accuracy of CCTA measurements. The systematic underestimation of lumen area by CCTA may be influenced by the degree of coronary calcification, and that the absence of uniformly available Agatston scores precluded stratified analysis by calcium burden. This limitation and its potential impact on measurement accuracy. While our analysis aimed to account for demographic factors, the influence of specific image quality variations (e.g., motion and severe calcification) on measurement accuracy remains a challenge inherent to CCTA.

## 5 Conclusion

CCTA-derived left main cross-sectional area shows strong anatomical correlation with IVUS derived minimum lumen area and demonstrates high diagnostic performance for identifying IVUS defined significant LMCA stenosis in this Iranian cohort. The proposed CCTA threshold, derived in this Iranian cohort, may assist in non-invasive anatomical risk stratification; however, these findings should be interpreted in the context of anatomical assessment rather than direct functional significance. Larger, external validation studies incorporating physiological reference standards are warranted before broad clinical adoption.

## CRediT authorship contribution statement

**Golnaz Houshmand:** Writing – review & editing, Writing – original draft, Supervision, Investigation, Conceptualization. **Amirafraz Fallah Najmabadi:** Writing – review & editing, Investigation. **Pooya Eini:** Writing – review & editing, Writing – original draft, Formal analysis. **Mahdi Zahedi:** Writing – review & editing, Writing – original draft, Investigation. **Marzieh Motevalli:** Writing – review & editing, Investigation. **Mohammad Javad Alemzadeh-Ansari:** Writing – review & editing, Investigation.

## Declaration of Generative AI and AI-assisted technologies in the writing process

In the preparation of this article, the authors utilized the Grammarly application to enhance linguistic accuracy and clarity. The manuscript underwent meticulous double-checking to ensure precision, and the authors assume full responsibility for the integrity and originality of the content presented herein.

## Ethics approval and consent to participate

This retrospective study was approved by the institutional ethics committee (code: IR.RHC.REC.1404.219) and performed in line with the principles of the Declaration of Helsinki. The need for informed consent was waived by the Rajaie Cardiovascular Ethics Committee due to the retrospective nature of the study.

## Consent for publication

Not applicable.

## Statement for studies involving humans / animals

This retrospective study was approved by the institutional ethics committee (code: IR.RHC.REC.1404.219) and performed in line with the principles of the Declaration of Helsinki. The need for informed consent was waived by the Rajaie Cardiovascular Ethics Committee due to the retrospective nature of the study.

## Clinical trial number

Not applicable.

## Funding

None.

## Declaration of Competing Interest

The authors declare that they have no known competing financial interests or personal relationships that could have appeared to influence the work reported in this paper.

## Data Availability

The datasets generated during and/or analysed during the current study are available from the corresponding author on reasonable request.
